# Safety and efficacy of a sublingual-oral desensitization protocol in cow’s milk allergy treatment

**DOI:** 10.1186/2045-7022-3-S3-P24

**Published:** 2013-07-25

**Authors:** S Piedade, G Sampaio, Â Gaspar, C Arêde, LM Borrego, C Santa-Marta, M Morais-Almeida

**Affiliations:** 1 Immunoallergy Department, CUF Descobertas Hospital, Lisbon, Portugal

## Background

Food allergies have become more prevalent and long lasting over the past two decades, namely cow’s milk allergy (CMA). Standard management for this disease is based on allergen avoidance and symptomatic treatment of accidental allergic reactions. The possibility of obtaining oral desensitization in patients with food allergy is still a mater of debate but seems to be a promising specific approach to modify the prognosis.

## Methods

In order to document and share experiences we present a protocol of sublingual-oral desensitizing treatment (SODT) applied, from May 2009 to July 2012, to 27 children with IgE-mediated CMA. The protocol, using pure CM as allergen extract, began with sub-lingual doses followed by oral ingestion of increasing doses of CM, always in Hospital settings, until reaching the target dose of 200mL/day. Informed consent was obtained at the beginning and at all treatment sessions, and the telephone number of the medical staff was offered.

## Results

Children had a mean age of 7.8 ± 4.4 years (1.5 to 16 years) and gender ratio M/F was 1.5:1. Personal history of sensitisation to common aeroallergens was present in about 80% of patients; all had allergic rhinitis and/or asthma as co-morbidity. Overall, in 5 Day-Hospital sessions, all the children achieved the daily intake of more than 200mL. During the SODT 19 children had mild to moderate adverse reactions, all successfully treated with oral anti-histamines and/or steroids. Severe reactions occurred in 2 cases: 1 had anaphylaxis after exercise, dependent on the intake of CM; other had anaphylaxis during the early induction stage by CM accidental ingestion, treated with adrenaline.

## Conclusion

Although randomized trials are needed, SODT may represent an alternative approach in children with CMA. Advantages of this SODT protocol are its safety and efficacy, dramatically reducing the risk of severe reactions after inadvertent ingestion of the allergen and improving the quality of life of these patients and their family.

## Disclosure of interest

None declared.

